# Procedure for Determining the Uncertainties in the Modeling of Surface Roughness in the Turning of NiTi Alloys Using the Monte Carlo Method

**DOI:** 10.3390/ma13194338

**Published:** 2020-09-29

**Authors:** Małgorzata Kowalczyk, Krzysztof Tomczyk

**Affiliations:** 1 Mechanical Faculty, Cracow University of Technology, Jana Pawła II 37 Avenue, 31-864 Krakow, Poland; kowalczyk@mech.pk.edu.pl; 2 Faculty of Electrical and Computer Engineering, Cracow University of Technology, Warszawska 24 Street, 31-155 Krakow, Poland

**Keywords:** surface roughness, NiTi alloy, Monte Carlo method

## Abstract

The paper presents a procedure for the determination of uncertainties in the modeling of surface roughness in the turning of NiTi alloys. The presented procedure is applicable both to the analysis of the measurement values of the two main roughness factors, as well as to research related to the prediction and optimization of the machining process. Type A and B, total, and expanded uncertainties were considered herein, and the obtained uncertainty values were assessed. A procedure for optimizing machining by applying the Monte Carlo (MC) method is also presented. The solutions presented in this paper are important from the point of view of practical solutions related to the prediction and optimization of the machining process. The considered procedure for determining and assessing uncertainty can be useful for the optimal selection of both machining parameters and measuring tools.

## 1. Introduction

The dynamic development of science in the modern world is strongly correlated with the demand for more and more new construction materials with properties adapted to new technologies [1,2,3,4,5]. However, it should be remembered that the practical use of these materials is inextricably corresponded to the methods of their production and treatment. In order to manufacture new products from difficult-to-machine materials, such as shape memory alloys, titanium alloys, nickel alloys, and special ceramics, there is a need to search for more and more effective treatment methods that exceed technological barriers [6,7,8,9].

Modern machining processes, especially of difficult-to-cut materials, which are widely used in industry, should ensure the best possible quality, efficiency, economy, reliability, and environmental friendliness of products [1,3,10].

Shape memory TiNi alloys are materials with unique functional properties, but at the same time have very favorable parameters in terms of mechanical strength, fatigue strength, specific weight, resistance to corrosion and aggressive environments, etc. For this reason, there is a great interest in these materials in the industry of modern instruments or mechanical systems used in technology and medicine [11,12,13,14,15]. However, in order to maintain their high position as construction materials, the costs of their treatment should be reduced. The problems associated with the machining of these alloys do not allow for higher cutting parameters to be achieved, and thus impede an increase in the machining efficiency while maintaining the appropriate quality of the surface layer. The main problems in the machining of TiNi alloys are related to high cutting temperatures and rapid tool wear, poor surface quality and cutting process efficiency, burr formation, and continuous chip, which is difficult to control [8,13,15,16,17,18,19,20,21,22,23].

Thus far, the priority in the industry has been to obtain the high-quality parameters of products made of NiTi alloys. The second in the hierarchy is the cost of their production. It can be expected that, in the future, due to the need to reduce and optimize production costs, research efforts will be directed toward the processes of shaping NiTi alloy elements using methods that ensure maximum efficiency while maintaining a good quality of the machined surface [6,11,12,13].

Due to the high requirements for elements made of NiTi alloys and the simultaneous need for effective machining of materials, the need to optimize the processes of such machining is evident. Despite the great advancement of research in the field of forecasting and optimization of the various machinability indices (surface roughness, tool wear, etc.) in relation to the processing of various construction materials, the literature lacks or highlights difficulties in finding items for which a detailed assessment of the issues related to prediction and optimization of surface roughness in the process of turning shape memory alloys has been carried out [7].

The guidelines for NiTi alloy machining presented in the scientific literature, which are based on the results of fragmentary experimental studies, are usually insufficient. In industry, cutting parameters are very often selected based on the experience of machine operators and programmers and on the recommendations of tool manufacturers in order to obtain an appropriate quality of the machined surface.

The machining process is characterized by a large number of parameters that affect it, which means that achieving optimal process efficiency while maintaining the required product quality is impossible, even for a highly qualified employee. The most unfavorable effects of this approach are the deterioration of product quality, an increase in operating costs and treatment time, a decrease in productivity, etc. [1,2,3,6,24].

Surface roughness is a measure of the technological quality of a product and a factor that has a large impact on the cost of production. Achieving the desired roughness value is a repetitive and empirical process that can be very time consuming. The mechanism of surface roughness formation is also very complicated and process-dependent; therefore, it is very difficult to calculate its value by using an analytical formula. The various theoretical models proposed in the relevant literature are not sufficiently accurate and are only applicable to a limited range of processes and cutting conditions, or must be used in connection with complicated diagrams or statistical tables. Therefore, an appropriate procedure is needed that can allow the surface roughness value to be assessed prior to the machining of the material, while being easy to use in industry and helping to minimize the time and cost of treatment. Moreover, such a procedure could be used to determine the appropriate cutting conditions to obtain the specific surface roughness [3,24,25,26,27,28,29,30,31].

The ability to predict surface roughness before machining has attracted great interest from many scientists, being the main goals of many research studies. The prediction of surface roughness is currently determined by using various techniques such as theoretical models [3,32,33,34,35], response surface methodology (RSM) [3,6,9,36,37,38], the Taguchi procedure [3,6,28,38,39,40,41,42,43], multiple linear regression equations [44], the Monte Carlo (MC) method [7,24,33,43,44,45,46,47,48,49,50,51,52], artificial intelligence through the use of the artificial neural networks (ANNs) [1,3,26,29,30,53,54,55,56], genetic algorithms (GAs) [3,57], fuzzy logic (FL) [3,36,54,58,59,60,61], the decision tree (DT) method [62], and expert systems (ES) [3]. Many research works show the use of these methods in the forecasting and optimization of surface roughness [3]. Researchers usually do not use only one modeling approach in their works, but look for a mutual compilation of the above strategies [3,6,36,37,38,39,54,59]. The benefits of using surface roughness prediction methods include an increase in the productivity and competitiveness of the production process and a simultaneous reduction in the need to re-machine a material to meet technical requirements [3,9,10,24].

Analyzing the relevant literature regarding the prediction and optimization of machining processes, it can be easily noticed that the current trend is the use of ANNs, GAs, RSM, and the Taguchi procedure for these purposes. The authors of this study note that despite the many application possibilities of the MC method [46,47,48,49,50,51,52], its application for solving the problems related to the prediction and optimization of machining has not been given much attention in the literature. This comment also applies to the analysis and assessment of the uncertainties [63] related to the above-mentioned prediction and optimization procedures. The developed model of surface roughness prediction together with the uncertainty determination procedure can significantly reduce the cost of machining of shape memory alloys while maintaining the optimal quality of the machined surface.

Taking into account the above literature review, this paper presents a procedure for determining the uncertainties, together with their analysis, in the modeling of surface roughness in the turning of NiTi alloys by employing the MC method. For the purposes of applying the MC method, the pseudorandom number generator with a uniform distribution was used [64]. The procedure presented here makes it possible to easily evaluate the suitability of the obtained results in the field of machining and, therefore, to choose other solutions in the event of obtaining unsatisfactory uncertainty values.

## 2. General Assumptions

Experimental tests for the purpose of determining a mathematical model were carried out for the operation of precision turning of a 6.38 mm shaft, made of the shape memory alloy β-NiTi (nitinol) with the following chemical composition: 52.85 at.% Ni and 47.15 at.% Ti. Machining was carried out dry in an air atmosphere. A CCMT 060202 polycrystalline diamond (PCD) plate (Iscar, Tel Aviv-Yafo, Israel) was used for the treatment. The research plan to determine the impact of three independent factors, namely, feed (f (mm/rev)), depth of cut ($a_{p}$ (mm)), and cutting speed ($v_{c}$ (m/min)), on the values of the selected factors Sa and Sz (i.e., 3D areal surface texture parameters) of the surface roughness was developed according to the Taguchi experiment design guidelines (DOE) [6]. The factors Sa (i.e., the arithmetical mean height of the surface) and Sz (i.e., the maximum height of the surface—sum of the maximum peak height value and the maximum pit height value within a definition area) were defined in accordance with the standard ISO25178 [62].

Generally, Sa is represented by the equation [27]:

(1) $$Sa=\frac{1}{LB}\int_{0}^{L}\int_{0}^{B}\left|\eta \left(x,y\right)\right|dxdy$$

where η(x,y) is the deviation of the surface irregularities from the base plane, and L and B are the length and the width of the given section of surface corresponding to the baseline for the given type of surface irregularities, respectively.

The research plan is represented in the form of the so-called orthogonal table *L*9, which describes the individual research trials for the three factors with three different values, which are called the levels. The parameter values for the machining were selected on the basis of the generally available NiTi alloy tests. These values are: f= 0.038, 0.058, 0.077 mm/rev; $a_{p}$ = 0.03, 0.08, 0.13 mm; $v_{c}$ = 30, 40, 50 m/min [6]. The 3D roughness measurements of the NiTi-treated surface were performed by using the Taylor Hobson measurement system. To perform the surface roughness measurements, a measuring tip with a rounding radius of 2 μm was used, and the measurements were repeated ten times. The periodic nature of the surface was included for analysis, and as a result, the calculation of the selected parameters of topography was obtained. The cut-off value $\lambda_{c}$ of the filter was selected based on the values recommended for periodic profiles. The value $\lambda_{c}=0.8$ mm was selected [6] based on the obtained ranges of surface roughness.

The mathematical relationships between the input data (f, $a_{p}$, and $v_{c}$) and the output factors (Sa and Sz) for the Taguchi experiment were obtained here. The experimental basis for these relationships can be found in previous work [6], while the model for determining the factors Sa and Sz was appointed using RSM.

The analysis with the Taguchi method mentioned above is only for the main factors Sa and Sz without any consideration of the correlation between them. Therefore, herein, RSM-based regression was used for the analysis of the correlation between such factors, which revealed that the contour plots of Sa were represented by corresponding curves. Therefore, the mathematical model used for predicting the suitable value was the quadratics model:

(2) $$y=b_{0}+b_{1}x_{1}+b_{2}x_{2}+b_{3}x_{3}+b_{4}x_{4}+b_{11}x_{1}{}^{2}+b_{22}x_{2}{}^{2}+b_{33}x_{3}{}^{2}+b_{44}x_{4}{}^{2}+b_{12}x_{1}x_{2}+b_{13}x_{1}x_{3}+b_{14}x_{1}x_{4}+b_{23}x_{2}x_{3}+b_{24}x_{2}x_{4}+b_{34}x_{3}x_{4}$$

where $b_{0},$ $b_{1},$ $b_{2},$ $b_{3},$ $b_{4},$ $b_{11},$ $b_{22},$ $b_{33},$ $b_{44},$ $b_{12},$ $b_{13},$ $b_{14}$, $b_{23},$ $b_{24}$, and $b_{34}$ are constant values, while $x_{1},$ $x_{2},$ $x_{3}$, and $x_{4}$ are model parameters (i.e., input parameters).

Model (1) can be obtained by considering the full quadratics model represented by the mathematical models:

(3) $$Sa^{\mathrm{mod}}=2.046-2.436\cdot f-2.038\cdot a_{p}+0.08588\cdot v_{c}+43.2\cdot f^{2}+14.47\cdot a_{p}{}^{2}+0.001022\cdot v_{c}{}^{2}$$

and

(4) $$Sz^{\mathrm{mod}}=37.49-161.9\cdot f-34.25\cdot a_{p}-\;1.353\cdot v_{c}+1382\cdot f^{2}+184.7\cdot a_{p}{}^{2}+0.01612\cdot v_{c}{}^{2}$$

where

(5) $$v_{c}=\frac{\pi dn}{1000}$$

while d and n are the diameter of the machined surface and the number of rotations, respectively.

The factors $Sa^{\mathrm{mod}}$ and $Sz^{\mathrm{mod}}$ represent the arithmetical mean height and the maximum height of the surface (sum of the maximum peak height value and the maximum pit height value within a definition area), respectively. These factors describe the height or height distribution of the surface irregularities.

## 3. Results of the Measurements and Associated Uncertainties Obtained Experimentally

The predetermined values of parameters f, $a_{p}$ and n, for which the factors Sa and Sz were determined with a constant value of diameter d equal to 6.380 mm, are tabulated in Table 1 (rows 1, 2, …, 9).

Table 2 (columns *M*_s_ = 1, 2, …, 9) summarizes the series of measurements of the factors Sa and Sz, which were determined for particular rows from Table 1. Each series contains *N* = 10 measurements, while the rows described by $\bar{Sa}$ and $\bar{Sz}$ are the mean values of Sa and Sz determined on the basis of a particular *n* measurement. The mean $\bar{Sa}$ and $\bar{Sz}$ were adopted below as the value of the factors Sa and Sz.

The uncertainty of type A associated with the measurement of factors Sa and Sz is presented by the equation:

(6) $$u_{A}\left(x\right)=\sqrt{\frac{1}{N-1}\overset{N}{\underset{n=1}{\sum }}{\left(x_{n}-\bar{x}\right)}^{2}}$$

where

(7) $$\bar{x}=\frac{1}{N}\overset{N}{\underset{n=1}{\sum }}x_{n}$$

corresponds to the mean $\bar{Sa}$ and $\bar{Sz}$ [47,63].

The uncertainty of type B associated with the measurement of factors Sa and Sz is:

(8) $$u_{B}\left(x\right)=\Delta$$

where Δ=0.001 μm is the reading resolution of the above parameters.

The total and expanded uncertainties are determined based on the following formulas:

(9) $$u\left(x\right)=\sqrt{u_{A}{}^{2}\left(x\right)+u_{B}{}^{2}\left(x\right)}$$

and

(10) $$U\left(x\right)=k_{p}u\left(x\right)$$

respectively, where $k_{p}$ is the coverage factor and for a distribution with a confidence level of 0.95 equal to 2 [47,63].

The combined uncertainties associated with the factors $Sa^{\mathrm{mod}}$ i $Sz^{\mathrm{mod}},$ which are determined based on Equations (3) and (4), are defined by the formulas:

(11) $$u_{c}\left(Sa^{\mathrm{mod}}\right)=\sqrt{{\left[\frac{\partial S_{a}{}^{m}}{\partial f}u_{B}\left(f\right)\right]}^{2}+{\left[\frac{\partial S_{a}{}^{m}}{\partial a_{p}}u_{B}\left(a_{p}\right)\right]}^{2}+{\left[\frac{\partial S_{a}{}^{m}}{\partial v_{c}}u_{c}\left(v_{c}\right)\right]}^{2}}$$

and

(12) $$u_{c}\left(Sz^{\mathrm{mod}}\right)=\sqrt{{\left[\frac{\partial S_{z}{}^{m}}{\partial f}u_{B}\left(f\right)\right]}^{2}+{\left[\frac{\partial S_{z}{}^{m}}{\partial a_{p}}u_{B}\left(a_{p}\right)\right]}^{2}+{\left[\frac{\partial S_{z}{}^{m}}{\partial v_{c}}u_{c}\left(v_{c}\right)\right]}^{2}}$$

where $u_{B}\left(f\right)$ and $u_{B}\left(a_{p}\right)$ are the uncertainties of type B associated with the parameters f and $a_{p},$ while $u_{c}\left(v_{c}\right)$ is the combined uncertainty associated with the parameter $v_{c}$ and is determined based of the following formula:

(13) $$u_{c}\left(v_{c}\right)\;=\sqrt{{\left[\frac{\partial v_{c}}{\partial d}u_{B}\left(d\right)\right]}^{2}+{\left[\frac{\partial v_{c}}{\partial n}u_{B}\left(n\right)\right]}^{2}}$$

where $u_{B}\left(d\right)$ and $u_{B}\left(n\right)$ [63] are the uncertainties of type B related to determination of the diameter d and the number of rotations n, respectively. The uncertainties $u_{B}\left(f\right),$ $u_{B}\left(a_{p}\right),$ $u_{B}\left(d\right)$, and $u_{B}\left(n\right)$ are equal to 0.001 mm/rev, 0.01 mm, 0.01 mm, and 1 rev, respectively. These uncertainties result from the resolution of the measurement devices used.

The expanded uncertainties are determined based on the following formula:

(14) $$U\left(y\right)=k_{p}u_{c}\left(y\right)$$

where y corresponds to the values Sa and Sz, while $k_{p}=2.$

The mean values $\bar{Sa}$ and $\bar{Sz}$ of the measured factors Sa and Sz as well as the uncertainty determined on the basis of the Equation (10) are tabulated in Table 3. The uncertainties: $u_{A}\left(\bar{Sa}\right)$ and $u\left(\bar{Sa}\right)$ are omitted in this Table because they are equal to each other and are exactly half of the value of the uncertainty $U\left(\bar{Sa}\right)$ for all rows. The situation is analogical in the case of uncertainties $u_{A}\left(\bar{Sz}\right)$ and $u\left(\bar{Sz}\right)$. Hence, only the uncertainty $U\left(\bar{Sz}\right)$ is included in Table 3.

The values of each row from Table 3 correspond to the parameters from the particular rows (i.e., the different combinations of the parameters f, $a_{p}$, and *n*) of Table 1.

The uncertainties related to the parameters $\bar{Sa}$ and $\bar{Sz}$ refer only to their last significant numbers (zeros at the beginning are not taking into account as significant numbers; hence, only the last number greater than zero is included as a significant one), while the uncertainties related to the factor $Sa^{\mathrm{mod}}$ refer to one or two of the last significant number/s. The uncertainties related to the factor $Sz^{\mathrm{mod}}$ refer to the last two or three significant number. For each case under consideration, the uncertainties associated with the factors obtained on the basis of the mathematical models are from several to several times higher than the uncertainties determined for the values of the parameters obtained on the basis of the measurement data. In order to reduce the uncertainty value associated with the mathematical models, it is necessary to increase the resolution value of the measuring devices applied and to use a more precise method for determining these models.

## 4. Modeling of Surface Roughness

Below, the modeling procedure of surface roughness based on the mathematical models given by Equations (3) and (4) and by employing the MC method, respectively, are presented in the Section 4.1 and Section 4.2 . The values of parameters Sa and Sz and associated uncertainties are determined there.

### 4.1. Calculations Based on the Mathematical Models

The values of the functions $Sa^{\mathrm{mod}}$ and $Sz^{\mathrm{mod}}$ determined based on Equations (3) and (4), as well as the associated uncertainties determined on the basis of the Equations (11)–(14) are tabulated in Table 4.

Table 4 shows that for rows 1, 5, and 9, the values of the factors $Sa^{\mathrm{mod}}$ and $Sz^{\mathrm{mod}}$ obtained on the basis of mathematical models (3) and (4) have lower values than the values of $\bar{Sa}$ and $\bar{Sz}$ obtained from the measurements. These differences are equal to a maximum of 20% and are related to the inaccuracy of determining models (3) and (4).

### 4.2. Modeling Based on the Monte Carlo Method

The applied MC method uses a pseudo-random number generator with the uniform distribution U(0, 1) according to the Wichmann–Hill algorithm with a period equal to $2^{121}.$ The pseudo-random number generation procedure is performed using the following six main steps [47,49,64]:

1. Create four vectors that include the integer numbers:
  (15) A=[11600  47003  23000  33000],  B=[185127  45688  93368  65075],C=[10379  10479  19423  8123],D = [P+456  P+420  P+300  P]
  where P= 247,483,123.
2. Set the four-element initial vector **W** from the range of 0 to $2^{31}-1.$
3. Calculate:
  (16) $$W_{i}=A_{i}\left[W_{i}\cdot mod\left(B_{i}\right)\right]-C_{i}\cdot floor\left(W_{i}/B_{i}\right),\mathrm{for}\;i=0,\ldots ,\;3$$
  where $A_{i},$ $B_{i},\;C_{i}$, and $W_{i}$ denote the particular elements of the vectors A, B, C, and **W**, while the functions mod(x) and floor(x) denote the modulo from x and the largest integer digit no greater than x, respectively.
4. If $W_{i}<0,$ replace $W_{i}$ with $W_{i}+D_{i}.$
5. Calculate:
  (17) $$S=\sum_{i=1}^{3}W_{i}/B_{i}$$
6. Finally, return:
  (18) U=S−floor(S)

For 64-bit computers, Equation (16) can be replaced by the following simple formula:

(19) $$W_{i}=A_{i}W_{i}\cdot mod\left(D_{i}\right)$$

**Figure 1:**
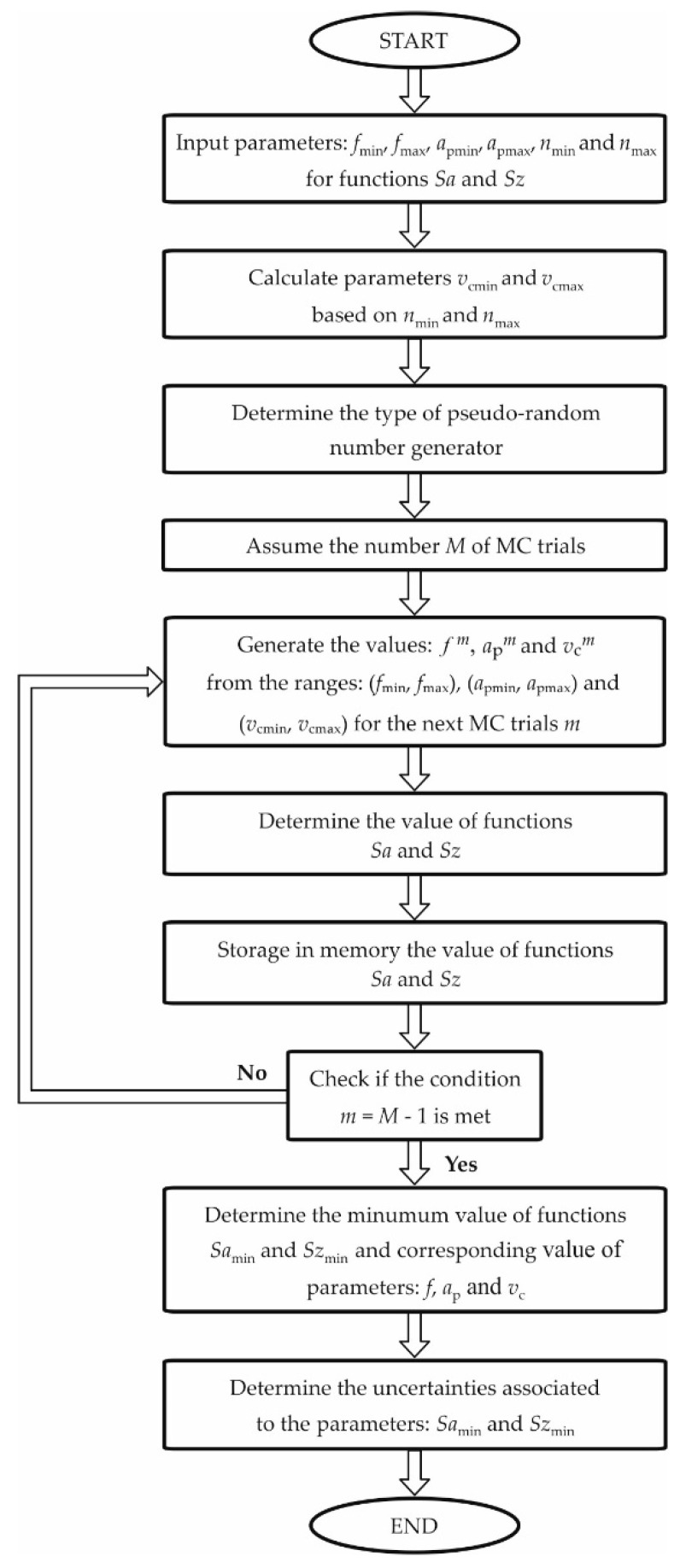
Block diagram of the applied MC method.

Figure 1 shows a block diagram of the applied MC method for determining the optimal (minimum) values of the factors (functions) Sa and Sz.

The four cases with the number M of MC trials equal to $10^{3}$, $10^{4}$, $10^{5}$, and $10^{6}$ are checked below.

Uncertainties associated with the parameters Sa and Sz are determined based on the formula [49]:

(20) $$u\left(z\right)=\sqrt{\frac{1}{M-1}\overset{M-1}{\underset{m=0}{\sum }}{\left(z_{m}-\bar{z}\right)}^{2}}$$

where

(21) $$\bar{z}=\frac{1}{M}\overset{M-1}{\underset{m=0}{\sum }}z_{m}$$

and z corresponds to the parameters Sa and Sz.

Table 5 summarizes the results of the calculations of the minimum values (rows 2 and 9) of the functions described by Equations (3) and (4) and defined by $Sa^{MC}{}_{min}$ and $Sz^{MC}{}_{min}$ for the case of five different numbers of MC draws (the first row): $10^{3},$ $10^{4},$ $10^{5},$ $10^{6}$, and $10^{7}$. For the values of the functions $Sa^{MC}{}_{min}$ and $Sz^{MC}{}_{min}$ for all Monte Carlo trials, the corresponding drawing number m (rows 3 and 10), the values of parameters f, $a_{p}$, and *n* (rows 4–6 and 11–13), the mean values of ${\bar{Sa}}^{MC}$ and ${\bar{Sz}}^{MC}$ (rows 7 and 14), and the associated uncertainties (rows 8 and 15) are determined.

The analogous minimum values of factors $Sa^{MC}{}_{min}$ and $Sz^{MC}{}_{min}$ and the analogous uncertainty values of $u\left({\bar{Sa}}^{MC}\right)$ and $u\left({\bar{Sz}}^{MC}\right)$ for the number of MC draws are equal to $10^{5}$ and $10^{6}.$ The values $Sa^{MC}{}_{min}=0.140\;\mu m$ and $Sz^{MC}{}_{min}=2.770\;\mu m$ are assumed to be the optimal solutions for the MC method.

In comparison with Table 3 and Table 4, Table 5 includes the following parameters: f, $a_{p}$ and n for which the lowest (optimal) values of the parameters Sa and Sz are obtained.

Table 3 and Table 4 includes the values $\bar{Sa}$, $\bar{Sz}$, $Sa^{\mathrm{mod}}$ and $Sz^{\mathrm{mod}}$ as well as the associated extended uncertainties: $U\left(\bar{Sa}\right),$ $U\left(\bar{Sz}\right),$ $U\left(Sa^{\mathrm{mod}}\right)$ and $U\left(Sz^{\mathrm{mod}}\right)$ which are determined based on the measurement data and mathematical models (index: mod) for nine cases of parameters: f, $a_{p}$, and n which are included in Table 1. The parameters, $a_{p}$ and n were selected based on the Taguchi procedure. However, the results obtained for them are not optimal parameters for machining process. Only the parameters f, $a_{p}$ and n, determined on the basis of the MC method and summarized in Table 5, provide the minimum (optimal) values of the parameters Sa and Sz. By extending the uncertainty $u\left({\bar{Sa}}^{MC}\right)$ from Table 5 according to Equation (10) and for MC equal to 10^5^ or 10^6^, we obtain $U\left({\bar{Sa}}^{MC}\right)$ = 0.006. Both uncertainties $u\left({\bar{Sa}}^{MC}\right)$ and $U\left({\bar{Sa}}^{MC}\right)$ are close (referring to the last significant number) to the nine cases tabulated in Table 4. The case is quite different for uncertainty $u\left({\bar{Sz}}^{MC}\right)$ contained in Table 5, where after expansion the values of uncertainty $U\left({\bar{Sz}}^{MC}\right)$ are equal to 0.850 and 0.852, respectively for MC = 10^5^ or MC = 10^6^ Both uncertainties $u\left({\bar{Sz}}^{MC}\right)$ and $U\left({\bar{Sz}}^{MC}\right)$ have 2–8 times higher values than those summarized in Table 4. This means that for the parameter *Sz*, higher uncertainties are obtained than for those listed in Table 4. On the other hand, the values of the parameters: *f*, *a*_p_ and n which are related to these uncertainties are the optimal solution for prediction of the machining process.

**Figure 2:**
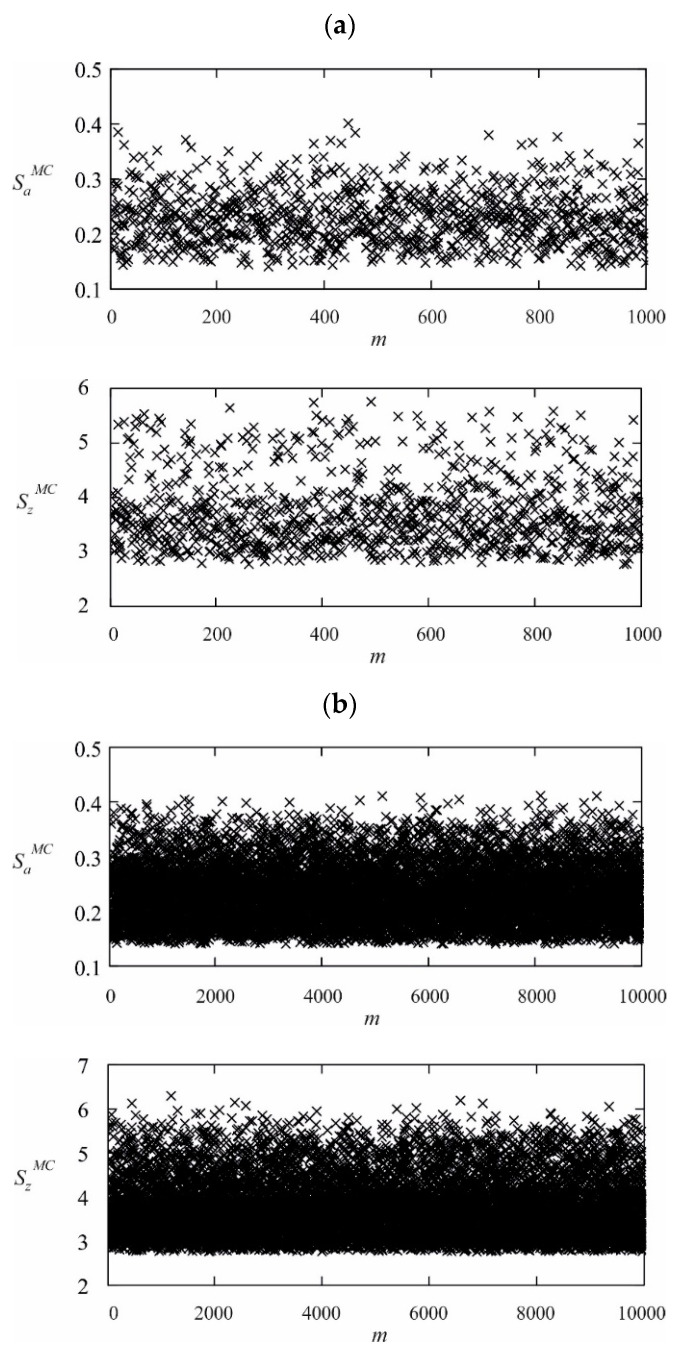
(**a**) Values of factors $Sa^{MC}$ and $Sz^{MC}$ for the Monte Carlo trials equal to $10^{3}$. (**b**) Values of factors $Sa^{MC}$ and $Sz^{MC}$ for the Monte Carlo trials equal to $10^{4}$.

Figure 2a,b show the values of factors $Sa^{MC}$ and $Sz^{MC}$ for the particular Monte Carlo trials m and for the total MC draw equal to 10^3^ and $10^{4},$ respectively. There are no figures for the number of MC draws equal to 10^5^ and $10^{6}$ due to their lack of legibility (i.e., their high concentration of random points).

The above figures show a higher concentration of random points corresponding to the values of the functions $Sa^{MC}$ and $Sz^{MC}$ toward their minimum values. An analogous distribution of points was obtained for MC draws equal to 10^5^ and 10^6^. The reason is the syntax of the functions $Sa^{MC}$ and $Sz^{MC},$ which, for a generator with a uniform distribution, provides values for these points that are close to the minimum.

## 5. Conclusions

The procedure for determining the uncertainties related to the roughness parameters presented in this paper allow for easy and quick evaluation of the obtained results related to prediction and optimization (by using the MC method) in the machining process. The results show that the uncertainties, in comparison to the measured values, have values a dozen times higher for models (3) and (4) that were determined in previous works. The reason for this is the fact that the accuracy (uncertainties) of these models was not tested in these works.

Increasing the accuracy of such models can be achieved by using RSM based on, e.g., the radial basis functions (RBFs) determined by using an ANN. In a simpler case, it is possible to use approximation polynomials with an appropriately selected order, controlled by the determination of the associated uncertainty of such modeling. For such accurate models, it is possible to use the MC method for the optimization and prediction of the machining process, as shown in this paper.

The results obtained in this paper highlight the need for a more precise determination of the factors Sa and Sz in future works. Thanks to such precise models, the value of type A uncertainty and the corresponding value of expanded uncertainty can be reduced in the optimization and prediction process of machining. An additional reduction of the expanded uncertainty is possible by reducing the value of the type B uncertainty, which can be fulfilled by increasing the resolution of the tools dedicated to machining or the resolution of the measurement devices used.

## Figures and Tables

**Table 1:** Values of the parameters, $a_{p}$, and *n*.

no.	f [mm/rev]	$a_{p}$ [mm]	$v_{c}$ [m/min]	*n* [rev/min]
1	0.038	0.030	20	1498
2		0.080	30	1997
3		0.130	50	2496
4	0.058	0.030	20	1997
5		0.080	30	2496
6		0.130	50	1498
7	0.077	0.030	20	2496
8		0.080	30	1498
9		0.130	50	1997

**Table 2:** Measurement series *M*_s_ of the factors and *Sa* and Sz.

	*M*_s_	1	2	3	4	5	6	7	8	9
*n*										
		Factor *Sa* [μm]								
1		0.454	0.179	0.288	0.244	0.333	0.427	0.261	0.430	0.407
2		0.404	0.159	0.278	0.194	0.363	0.377	0.351	0.380	0.387
3		0.304	0.039	0.148	0.094	0.383	0.277	0.231	0.280	0.287
4		0.284	0.049	0.128	0.074	0.223	0.257	0.231	0.260	0.307
5		0.424	0.169	0.288	0.214	0.323	0.397	0.351	0.400	0.407
6		0.414	0.159	0.258	0.204	0.313	0.387	0.361	0.390	0.387
7		0.344	0.089	0.188	0.134	0.293	0.317	0.271	0.320	0.307
8		0.444	0.189	0.288	0.234	0.313	0.417	0.371	0.420	0.427
9		0.314	0.059	0.138	0.104	0.273	0.287	0.261	0.290	0.317
10		0.354	0.099	0.178	0.144	0.213	0.327	0.321	0.330	0.337
$\bar{Sa}$		0.374	0.119	0.218	0.164	0.303	0.347	0.301	0.350	0.357
		**Factor *Sz* [μm]**								
1		6.921	3.390	4.267	3.183	4.390	5.338	4.940	5.203	4.087
2		6.845	3.312	4.186	3.105	4.341	5.197	4.861	5.081	4.005
3		6.950	3.419	4.313	3.212	4.432	5.296	4.979	5.261	4.119
4		6.864	3.346	4.214	3.129	4.327	5.219	4.882	5.140	4.036
5		6.936	3.371	4.276	3.197	4.301	5.321	5.021	5.212	4.105
6		6.831	3.340	4.209	3.083	4.305	5.181	4.856	5.114	4.004
7		6.915	3.387	4.261	3.171	4.360	5.348	4.937	5.223	4.042
8		6.963	3.392	4.312	3.226	4.412	5.189	4.867	5.243	4.137
9		6.970	3.402	4.321	3.225	4.392	5.317	5.005	5.260	4.148
10		6.833	3.275	4.184	3.112	4.261	5.158	4.894	5.081	4.024
$\bar{Sz}$		6.903	3.363	4.254	3.164	4.352	5.256	4.924	5.179	4.071

**Table 3:** Mean $\bar{Sa}$ and $\bar{Sz}$ and the associated uncertainties.

no.	Mean $\bar{Sa}$ $\mathrm{and}\;\bar{Sz}$		Uncertainties Based on the Measurement Data	
	$\bar{Sa}$	$\bar{Sz}$	$U\left(\bar{Sa}\right)$	$U\left(\bar{Sz}\right)$
	[μm]			
1	0.374	6.903	0.008	0.006
2	0.119	3.363	0.006	0.004
3	0.218	4.254	0.010	0.006
4	0.164	3.164	0.008	0.006
5	0.303	4.352	0.006	0.006
6	0.347	5.256	0.008	0.012
7	0.301	4.924	0.008	0.008
8	0.350	5.179	0.008	0.012
9	0.357	4.071	0.006	0.006

**Table 4:** Values of the functions $Sa^{\mathrm{mod}}$ and $Sz^{\mathrm{mod}}$ and the associated uncertainties.

no.	Values of the Functions $Sa^{\mathrm{mod}}$$\;\mathrm{and}\;Sz^{\mathrm{mod}}$		Uncertainties Based on the Mathematical Models			
	$Sa^{\mathrm{mod}}$	$Sz^{\mathrm{mod}}$	$u_{c}\left(Sa^{\mathrm{mod}}\right)$	$U\left(Sa^{\mathrm{mod}}\right)$	$u_{c}\left(Sz^{\mathrm{mod}}\right)$	$U\left(Sz^{\mathrm{mod}}\right)$
	[μm]					
1	0.311	6.381	0.018	0.036	0.239	0.478
2	0.145	3.446	0.003	0.006	0.074	0.148
3	0.257	4.660	0.017	0.034	0.150	0.300
4	0.202	3.558	0.012	0.024	0.232	0.464
5	0.241	3.848	0.004	0.008	0.051	0.102
6	0.373	5.326	0.018	0.036	0.139	0.278
7	0.328	5.04	0.013	0.026	0.238	0.476
8	0.387	5.568	0.005	0.010	0.072	0.144
9	0.294	3.557	0.018	0.036	0.147	0.294

**Table 5:** Calculation results of the minimum value of Equations (3) and (4) by using the MC method.

no.	Parameters/Factors	Results			
1	MC [no.]	$10^{3}$	$10^{4}$	$10^{5}$	$10^{6}$
2	$Sa^{MC}{}_{min}$ [μm]	0.141	0.141	0.140	0.140
3	m [no.]	294	8144	60915	692749
4	f [mm/rev]	0.039	0.039	0.038	0.038
5	$a_{p}$ [mm]	0.070	0.075	0.071	0.070
6	*n* [rev/min]	2063	2123	2102	2101
7	${\bar{Sa}}^{MC}$ [μm]	0.230	0.230	0.230	0.230
8	$u\left({\bar{Sa}}^{MC}\right)$ [μm]	0.002	0.003	0.003	0.003
9	$Sz^{MC}{}_{min}$ [μm]	2.780	2.777	2.770	2.770
10	m [no.]	966	6276	87578	179181
11	f [mm/rev]	0.057	0.060	0.059	0.059
12	$a_{p}$ [mm]	0.092	0.092	0.092	0.093
13	*n* [rev/min]	2061	2065	2098	2095
14	${\bar{Sz}}^{MC}$ [μm]	3.712	3.725	3.733	3.730
15	$u\left({\bar{Sz}}^{MC}\right)$ [μm]	0.425	0.417	0.425	0.426

## References

[1] Abbas A.T., Sharma N., Anwar S., Hashmi F.H., Jamil M., Hegab H. (2019). Towards Optimization of Surface Roughness and Productivity Aspects during High-Speed Machining of Ti–6Al–4V. Materials.

[2] Alajmi M.S., Almeshal A.M. (2020). Prediction and Optimization of Surface Roughness in a Turning Process Using the ANFIS-QPSO Method. Materials.

[3] Benardos P.G., Vosniakos G.C. (2003). Predicting Surface Roughness in Machining: A Review. Int. J. Mach. Tools Manuf..

[4] Romanska-Zapala A., Bomberg M., Yarbrough D.W. (2019). Buildings with Environmental Quality Management: Part 4: A path to the future NZEB. J. Build. Phys..

[5] Yarbrough D.W., Bomberg M., Romanska-Zapala A. (2019). On the Next Generation of Low Energy Buildings. Adv. Build. Energy Res..

[6] Kowalczyk M. (2017). Application of Taguchi Method to Optimization of Surface Roughness during Precise Turning of NiTi Shape Memory Alloy. Proc. SPIE.

[7] Kowalczyk M. (2018). Application of the Monte Carlo Method for the Optimization of Surface Roughness during Precise Turning of NiTi Shape Memory Alloy. Proc. SPIE.

[8] Lin H.C., Lin K.M., Chen Y.C. (2000). A Study on the Machining Characteristics of TiNi Shape Memory Alloys. J. Mater. Process. Technol..

[9] Matras A. (2020). Research and Optimization of Surface Roughness in Milling of SLM Semi-Finished Parts Manufactured by Using the Different Laser Scanning Speed. Materials.

[10] Karkalos N.E., Galanis N.I., Markopoulos A.P. (2016). Surface Roughness Prediction for the Milling of Ti–6Al–4V ELI Alloy with the use of Statistical and Soft Computing Techniques. Measurement.

[11] Chaudhari R., Vora J.J., Patel V., Lacalle L.N.L., Parikh D.M. (2020). Surface Analysis of Wire-Electrical-Discharge-Machining-Processed Shape-Memory Alloys. Materials.

[12] Elahinia M.H., Hashemi M., Tabesh M., Bhaduri S.B. (2012). Manufacturing and Processing of NiTi Implants: A Review. Prog. Mater. Sci..

[13] Guo Y., Klink A., Fu C., Snyder J. (2013). Machinability and Surface Integrity of Nitinol Shape Memory Alloy. CIRP Ann. Manuf. Technol..

[14] Kaynak Y., Robertson S.W., Karacac H.E., Jawahir I.S. (2015). Progressive Tool-Wear in Machining of Room-Temperature Austenitic NiTi Alloys: The Influence of Cooling/Lubricating, Melting, and Heat Treatment Conditions. J. Mater. Process. Technol..

[15] Piquard R., Acunto A.D., Laheurte P., Dudzinski D. (2014). Micro-End Milling of NiTi Biomedical Alloys, Burr Formation and Phase Transformation. Prec. Eng..

[16] Biermann D., Kahleyss F., Krebs E., Upmeier T. (2011). A Study on Micro-Machining Technology for the Machining of NiTi: Five-Axis Micro-Milling and Micro Deep-Hole Drilling. JMEPEG.

[17] Dash B., Das M., Mahapatra T.R., Mishra D.A. (2019). Concise Review on Machinability of NiTi Shape Memory Alloys. Mat. Tod. Proc..

[18] Kaynak Y., Karacac H.E., Jawahir I.S. (2014). Surface Integrity Characteristics of NiTi Shape Memory Alloys Resulting from Dry and Cryogenic Machining. Procedia CIRP.

[19] Kaynak Y., Karaca H.E., Noebe R.D., Jawahir I.S. (2013). Tool Wear Analysis in Cryogenic Machining of NiTi Shape Memory Alloys: A Comparison of Tool Wear Performance with Dry and MQL Machining. Wear.

[20] Weinert K., Petzoldt V., Kotter D. (2004). Turning and Drilling of NiTi Shape Memory Alloys. CIRP Ann. Manuf. Technol..

[21] Weinert K., Petzoldt V. (2004). Machining of NiTi based Shape Memory Alloys. Mater. Sci. Eng..

[22] Weinert K., Petzoldt V. (2008). Machining NiTi Micro-Parts by Micro-Milling. Mater. Sci. Eng..

[23] Wu S.K., Lin H.C., Chen C.C. (1999). A Study on the Machinability of a Ti 49,6 Ni 50,4 Shape Memory Alloy. Mater. Lett..

[24] Madić M., Radovanović M. (2014). Possibility of using the Monte Carlo Method for Solving Machining Optimization Problems. Mech. Eng..

[25] Nguyen H.T., Hsu Q.C. (2016). Surface Roughness Analysis in the Hard Milling of JIS SKD61 Alloy Steel. Appl. Sci..

[26] Zagorski I., Kłonica M., Kulisz M., Łoza K. (2018). Effect of the AWJM Method on the Machined Surface Layer of AZ91D Magnesium Alloy and Simulation of Roughness Parameters Using Neural Networks. Materials.

[27] Aver’yanova I.O., Bogomolov D.Y., Poroshin V.V. (2017). ISO 25178 Standard for Three-Dimensional Parametric Assessment of Surface Texture. Russ. Eng. Res..

[28] Kolahan F., Manoochehri M., Hosseini A. (2011). Application of Taguchi Method and ANOVA Analysis for Simultaneous Optimization of Machining Parameters and Tool Geometry in Turning. Eng. Tech..

[29] Mia M., Dhar N.R. (2016). Prediction of Surface Roughness in Hard Turning Under High Pressure Coolant using Artificial Neural Network. Measurement.

[30] Nalbant M., Gokkaya H., Toktas I., Sur G. (2009). The Experimental Investigation of the Effects of Uncoated, PVD and CVD-Coated Cemented Carbide Inserts and Cutting Parameters on Surface Roughness in CNC Turning and its Prediction using Artificial Neural Networks. Rob. Comp. Integ. Manuf..

[31] Shankar N.V.S., Shankar H.R., Kumar N.P., Saichandu K. (2020). Process Parameter Optimization for Minimizing Vibrations and Surface Roughness During Turning EN19 Steel Using Coated Carbide Tool. Mater. Today Proc..

[32] Lu X., Zhang H., Jia Z., Feng Y., Liang S.Y. (2018). Floor surface roughness model considering tool vibration in the process of micro-milling. Int. J. Adv. Manuf. Technol..

[33] Feng Y., Hsu F.-C., Lu Y.-T., Lin Y.-F., Lin C.-T., Lin C.-F., Lu Y.-C., Lu X., Liang S.Y. (2020). Surface roughness prediction in ultrasonic vibration-assisted milling. J. Adv. Mech. Des. Syst. Manuf..

[34] Feng Y., Hung T.-P., Lu Y.-T., Lin Y.-F., Hsu F.-C., Lin C.-F., Lu Y.-C., Lu X., Liang S.Y. (2019). Surface roughness modeling in Laser-assisted End Milling of Inconel 718. Mach. Sci. Tech..

[35] Feng Y., Hung T.-P., Lu Y.-T., Lin Y.-F., Hsu F.-C., Lin C.-F., Lu Y.-C., Lu X., Liang S.Y. (2018). Inverse Analysis of Inconel 718 Laser-Assisted Milling to Achieve Machined Surface Roughness. Int. J. Prec. Eng. Manuf..

[36] Bhuyan R.K., Mohanty S., Routara B.C. (2017). RSM and Fuzzy Logic Approaches for Predicting the Surface Roughness during EDM of Al-SiCp MMC. Mater. Today Proc..

[37] Öztürk S., Kahraman M.F. (2019). Modeling and Optimization of Machining Parameters during Grinding of Flat Glass using Response Surface Methodology and Probabilistic Uncertainty Analysis based on Monte Carlo Simulation. Measurement.

[38] Prasath K.M., Pradheep T., Suresh S. (2018). Application of Taguchi and Response Surface Methodology (RSM) in Steel Turning Process to Improve Surface Roughness and Material Removal Rate. Mater. Today Proc..

[39] Chomsamutr K., Jongprasithporn S. (2012). Optimization Parameters of Tool Life Model using the Taguchi Approach and Response Surface Methodology. Int. J. Comput. Inf. Sci..

[40] Motorcu A.R. (2010). The Optimization of Machining Parameters Using the Taguchi Method for Surface Roughness of AISI 8660 Hardened Alloy Steel. J. Mech. Eng. Sci..

[41] Kuram E., Simsek B.T., Ozcelik B., Demirbas E., Askin S. Optimization of the Cutting Fluids and Parameters Using Taguchi and ANOVA in Milling. Proceedings of the World Congress on Engineering.

[42] Rama R.S., Padmanabhan G. (2012). Application of Taguchi Methods and ANOVA in Optimization of Process Parameters for Metal Removal Rate in Electrochemical Machining of Al/5%SiC composites. Int. J. Eng. Res. Appl..

[43] Kahraman M.F., Öztürk S. (2019). Experimental Study of Newly Structural Design Grinding Wheel Considering Response Surface Optimization and Monte Carlo Simulation. Measurement.

[44] Lu X., Wang F., Xue L., Feng Y., Liang S.Y. (2019). Investigation of material removal rate and surface roughness using multi-objective optimization for micro-milling of inconel 718. Ind. Lub. Tribo..

[45] Lu X., Wang X., Sun J., Zhang H., Feng Y. The Influence Factors and Prediction of Curve Surface Roughness in Micro-Milling Nickel-Based Superalloy. Proceedings of the ASME 2018 13th International Manufacturing Science and Engineering Conference.

[46] Kubisa S., Moskowicz S. (2007). A Study on Transitivity of Monte Carlo based Evaluation of the Confidence Interval for a Measurement Result. Pomiary Kontrola Autom..

[47] BIPM, IEC, IFCC, ILAC, ISO, IUPAC, IUPAP, OIML (2008). Evaluation of Measurement Data—Supplement 1 to the ‘Guide to the Expression of Uncertainty in Measurement’—Propagation of Distributions Using a Monte Carlo Method.

[48] Tomczyk K. (2019). Influence of Monte Carlo Generations Applied for Modelling of Measuring Instruments on Maximum Distance Error. Trans. Inst. Meas. Control.

[49] Tomczyk K. (2020). Monte Carlo-based Procedure for Determining the Maximum Energy at the Output of Accelerometers. Energies.

[50] Palenčár R., Sopkuliak P., Palenčár J., Ďuriš S., Suroviak E., Halaj M. (2017). Application of Monte Carlo Method for Evaluation of Uncertainties of ITS-90 by Standard Platinum Resistance Thermometer. Meas. Sci. Rev..

[51] Guimarães Couto P.R., Carreteiro Damasceno J., Pinheiro de Oliveira S., Chan V.W.K. (2013). Monte Carlo Simulations applied to Uncertainty in Measurement. Theory and Applications of Monte Carlo Simulations.

[52] Harris P.M., Cox M.G. (2014). On a Monte Carlo Method for Measurement Uncertainty Evaluation and its Implementation. Metrologia.

[53] Sanjeevi R., Nagaraja R., Krishnan B.R. (2020). Vision-based Surface Roughness Accuracy Prediction in the CNC Milling Process (Al6061) using ANN. Mater. Today Proc..

[54] Singh N.K., Singh Y., Kumar S., Sharma A. (2020). Predictive Analysis of Surface Roughness in EDM using Semi-Empirical, ANN and ANFIS Techniques: A Comparative Study. Mater. Today Proc..

[55] Dudzik M., Mielnik R., Wrobel Z. Preliminary Analysis of the Effectiveness of the use of Artificial Neural Networks for Modelling Time-Voltage and Time-Current Signals of the Combination Wave Generator. Proceedings of the International Symposium on Power Electronics, Electrical Drives, Automation and Motion (Speedam).

[56] Dudzik M., Stręk A.M. (2020). ANN Architecture Specifications for Modelling of Open-Cell Aluminum under Compression. Math. Probl. Eng..

[57] Gopan V., Wins L.D., Surendran A. (2018). Integrated ANN-GA Approach for Predictive Modeling And Optimization of Grinding Parameters With Surface Roughness As the Response. Mater. Today Proc..

[58] Barzani M.M., Zalnezhad E., Sarhan A.A.D., Farahany S., Ramesh S. (2015). Fuzzy Logic based Model for Predicting Surface Roughness of Machined Al–Si–Cu–Fe die Casting Alloy using Different Additives-turning. Measurement.

[59] Naresh C., Bose P.S.C., Rao C.S.P. (2020). ANFIS based Predictive Model for Wire EDM Responses involving Material Removal Rate and Surface Roughness of Nitinol Alloy. Mater. Today Proc..

[60] Tseng T.L.B., Konada U., Kwon Y.J. (2016). A Novel Approach to Predict Surface Roughness in Machining Operations using Fuzzy Set Theory. J. Comp. Design. Eng..

[61] Zuperl U., Cus F. (2016). Surface Roughness Fuzzy Inference System within the Control Simulation of end Milling. Prec. Eng..

[62] Barrios J.M., Romero P.E. (2019). Decision Tree Methods for Predicting Surface Roughness in Fused Deposition Modeling Parts. Materials.

[63] BIPM, IEC, IFCC, ILAC, ISO, IUPAC, IUPAP, OIML (2008). Evaluation of Measurement Data—Guide to the Expression of Uncertainty in Measurement.

[64] Wichmann B.A., Hill I.D. (2006). Generating Good Pseudo-Random Numbers. Comput. Stat. Data Anal..

